# Complete mitochondrial genome of the commensal scale worm, *Arctonoe vittata* (Grube, 1855) (Polychaeta: Polynoidae), collected from benthic habitat of the eastern coast of Korea

**DOI:** 10.1080/23802359.2021.1955771

**Published:** 2021-07-22

**Authors:** Jiseon Park, Jongwoo Jung, Kwang-Soo Kim, Taeseo Park

**Affiliations:** a Interdisciplinary Program of Ecocreative, Ewha Womans University, Seoul, Republic of Korea; b Department of Science Education, Ewha Womans University, Seoul, Republic of Korea; c Division of Animal Resources, National Institute of Biological Resources, Incheon, Republic of Korea; d Overseas Biological Resources Team, National Institute of Biological Resources, Incheon, Republic of Korea

**Keywords:** *Arctonoe vittata*, commensal, scale worm, complete mitogenome, Polynoidae

## Abstract

The complete mitogenome sequence of the commensal polynoid scale worm *Arctonoe vittata* was determined for the first time in the present study. The total length of the newly sequenced mitogenome was 15,125 bp, including 13 protein-coding genes, 2 rRNA genes, and 22 tRNA genes. The phylogenetic position of *A. vittata* was examined by maximum likelihood analysis using concatenated 13 protein-coding genes with 18 selected polychaete species. *Arctonoe vittata* was nested within the suborder Aphroditiformia and closely related to *Aphrodita australis* among the selected species. The newly determined mitogenome sequence will be useful for further phylogenetic and evolutionary studies of this group.

The polynoid scale worm *Arctonoe vittata* (Grube, 1855) is known to be commensally associated with various invertebrates such as gastropods, asteroids, holothuroids, and even with other tube-dwelling polychaetes (Hanley 1989; Ruff 1995; Park et al. 2016). There are three nominal species in the genus *Arctonoe* Chanberlin, 1920: *A. vittata*, *Arctonoe fragilis* (Baird, 1863), and *Arctonoe pulchra* (Johnson, 1897). However, *A. vittata* is the only species known to be distributed in northeastern Asian waters (Okuda 1936; Uschakov 1982; Imajima and Hartman 1964; Imajima 1998, 2001; Park et al. 2016).

Mitochondrial genes have been widely used in phylogenetic and evolutionary studies of metazoans (Zhang et al. 2018). However, despite their high species diversity, only about 90 complete polychaete mitogenomes have been published to date (Aguado et al. 2016; Seixas et al. 2017; Zhang et al. 2018). The aim of the present study was to determine the complete mitochondrial genome sequence of *A. vittata* for the first time.

A single specimen of *A. vittata* was extracted from the mantle cavity of the snowy limpet [*Niveotectura pallida* (Gould, 1859)] collected by SCUBA divers in the subtidal rocky zone (at the depth of 27 m) of the East Sea (38.372315N, 128.523263E, Goseong-gun, Gangwon-do, Korea). The specimen was immediately fixed in pure ethanol for genomic DNA extraction. Species identification was performed under a field stereomicroscope (EZ4 HD, Leica, Germany) based on Park et al. (2016). Genomic DNA was extracted from the small body tissue using the DNeasy Blood & Tissue kit (Qiagen, Hilden, Germany). The REPLI-g Mitochondrial DNA Kit (Qiagen, Hilden, Germany) was used for mitochondrial DNA amplification. Mitochondrial genome sequencing and library construction were conducted using the NovaSeq 6000 sequencing system (Illumina, San Diego, CA, USA) and Truseq DNA PCR-Free kit (Illumina, San Diego, CA, USA). Assembler and annotation tools, GetOrganelle (Jin et al. 2020) and Chlorobox (Tillich et al. 2017), were used, respectively. A voucher specimen was housed at the National Institute of Biological Resources (NIBR, http://www.nibr.go.kr/, Taeseo Park, polychaeta@gmail.com), Korea (NIBRIV0000810302).

The total length of the newly determined complete mitogenome of *A. vittata* was 15,125 bp (GenBank accession no. MZ131647). The mitogenome consisted of 37 encoded genes (including 13 protein-coding genes, 2 rRNA genes, and 22 tRNA genes). The overall nucleotide composition was 28.6% A, 18.0% C, 13.9% G, and 39.4% T, with a high A + T content (68.1%). Phylogenetic analysis was conducted to examine the phylogenetic position of *A. vittata* using the MEGA X software (Kumar et al. 2018). The tree was reconstructed by the maximum likelihood method using the GTR + G + I model with a bootstrap of 1000 replicates. Thirteen protein-coding genes were concatenated from 11 Nereidiformia species, one Aphroditiformia species, five Glyceriformia species, and one Eunicida species as an outgroup. As a result, *A. vittata* was grouped into the suborder Aphroditiformia and found to be closely related to *Aphrodita australis* Baird, 1865 (Figure 1).

**Figure 1.**
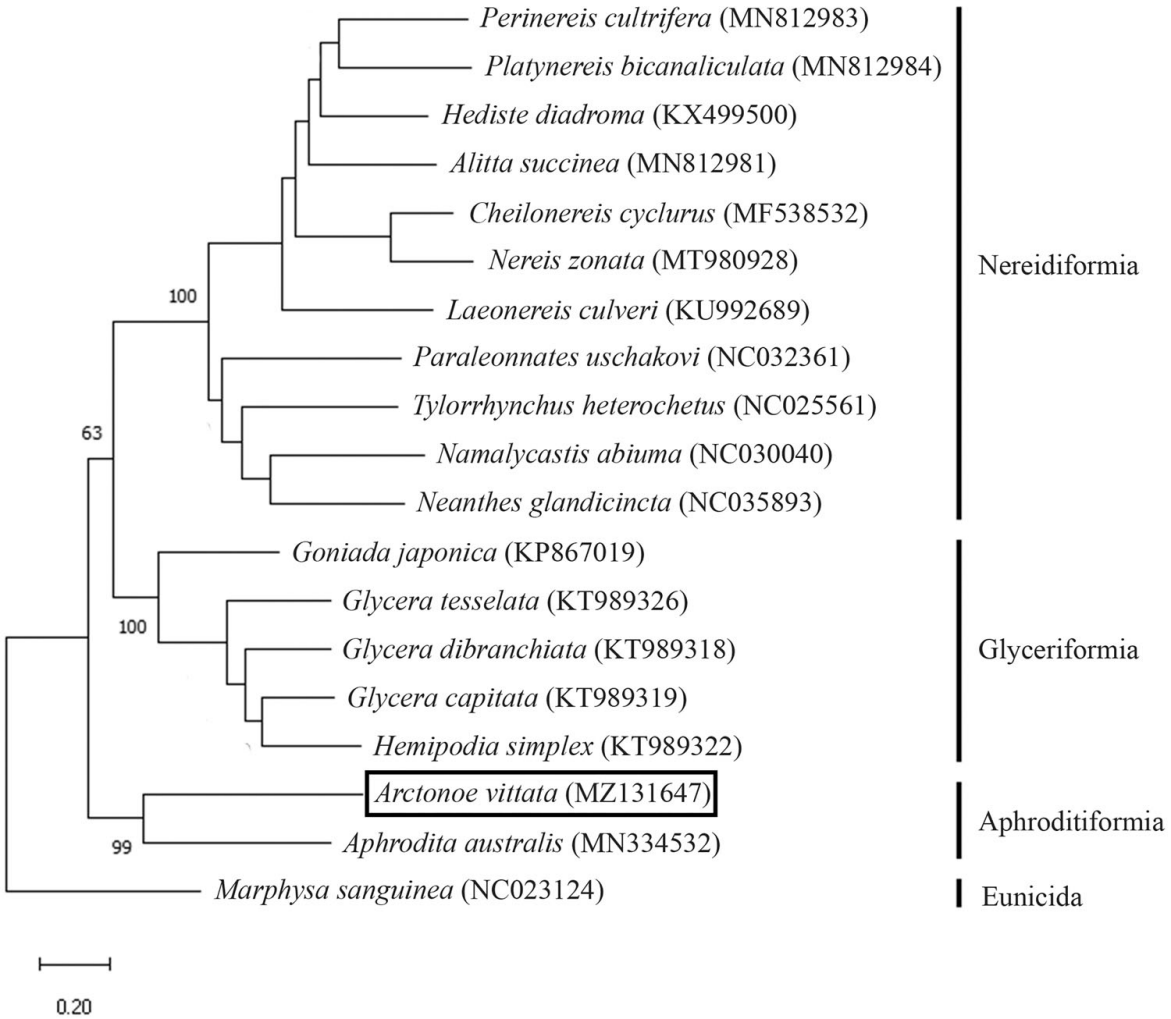
Maximum-likelihood (ML) tree reconstructed using a concatenated data set of 13 protein-coding genes based on 19 mitogenome sequences including *Arctonoe vittata* from the present study. Bootstrap replicates were performed 1000 times. The GenBank accession number of each species is shown in parentheses after the species name.

The newly determined mitogenome sequence of *A. vittata* will be useful for understanding the phylogenetic position of polynoid polychaetes.

## Data Availability

The genome sequence data that support the findings of this study are available in National Center for Biotechnology Information (NCBI) GenBank (https://www.ncbi.nlm.nih.gov) under the accession no. MZ131647. The associated BioProject, SRA, and BioSample numbers are PRJNA727905, SRR14565548, and SAMN19229861, respectively. The data that support the findings of this study are openly available in Mendeley Data (http://dx.doi.org/10.17632/67d3×6yyff.1).
